# Recycled Thermocol Nanofibers Based Smart Triboelectric Nanogenerators for AI‐Assisted Switching

**DOI:** 10.1002/smll.202512532

**Published:** 2026-02-18

**Authors:** Shubham V. Patil, Mahesh Y. Chougale, Thomas Cox, Sourabh Ghode, Abhishek Kulkarni, Parth Pandit, Niroshan Manoharan, Ajay Pandey, Jinho Bae, Deepak Dubal

**Affiliations:** ^1^ Centre For Materials Science School of Chemistry and Physics Queensland University of Technology Brisbane Australia; ^2^ Department of Ocean System Engineering Jeju National University Jeju South Korea; ^3^ School of Electrical Engineering and Robotics Queensland University of Technology Brisbane Australia

**Keywords:** EPS nanofibers, machine learning, smart system, TENG, waste recycling

## Abstract

The accumulation of non‐biodegradable thermocol (expanded polystyrene, EPS) waste presents a critical environmental challenge and an untapped resource for sustainable energy technologies. In this work, we demonstrate a waste‐to‐functional‐material strategy by transforming discarded thermocol into electrospun EPS nanofibers and employing them as an efficient tribonegative layer in a triboelectric nanogenerator (TENG). Unlike conventional polymer films, the nanofibrous EPS architecture introduces high surface roughness and electrospinning‐induced dipole alignment, leading to markedly enhanced charge generation. The resulting TENG delivers an open‐circuit voltage of ∼159 V, a short‐circuit current of ∼22 µA, and a maximum output powerdensity of 2083 mW/m^2^, outperforming widely used triboelectric polymers such as PTFE and PET. The device further exhibits excellent mechanical durability, long‐term stability over six months, and robust performance under varying humidity and temperature conditions. Beyond energy harvesting, the TENG is implemented as a self‐powered intelligent switching interface, where machine‐learning‐assisted signal processing enables reliable operation of a digital elevator system. This study establishes recycled EPS nanofibers as a high‐performance triboelectric material and demonstrates their potential for sustainable, intelligent, and self‐powered electronic systems.

## Graphical abstract

Waste expanded polystyrene is recycled into electrospun nanofibers for a sustainable triboelectric nanogenerator. The device exhibits enhanced surface morphology, improved triboelectric output, and multifunctional applications including energy harvesting, self‐powered sensing, and intelligent human‐machine interfaces using lightweight, flexible, and eco‐friendly materials.

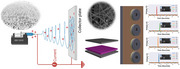

## 1 Introduction

As the demand for smart, autonomous, and low‐power electronic systems grows, particularly in applications like digital switching, smart sensing, and human‐machine interfaces, the need for sustainable and self‐powered technologies becomes increasingly critical [1, 2]. Triboelectric Nanogenerators (TENGs) have emerged as a key enabler in this space, offering an efficient method of converting low‐frequency mechanical energy into electrical signals [3]. These signals can be used not only for energy harvesting but also as real‐time triggers for interactive systems, such as touchless control panels, wearable electronics, and intelligent monitoring devices [4, 5]. Due to their high sensitivity, flexible design, and ability to function without an external power source [6, 7], TENGs are particularly well‐suited for digital switching applications in environments where hygiene, durability, or portability is essential, such as elevators, public kiosks, or industrial automation systems [8]. The efficiency and output performance of TENGs are largely governed by the selection of triboelectric materials and the surface morphology that influences charge density and contact area [9, 10, 11].

To enhance sustainability, recent studies have focused on the use of eco‐friendly or recycled materials, making TENGs an attractive solution not just technologically, but also environmentally [12, 13, 14]. In recent years, a variety of triboelectric nanogenerators based on recycled or waste‐derived materials have been reported, including rice paper, banana leaves, corn husk, coffee grounds, radish leaves, peanut shells, and other agricultural residues. These materials offer clear advantages in terms of renewability, biodegradability, and low environmental impact, making them attractive for sustainable energy harvesting. However, such bio‐derived triboelectric materials often suffer from inherent limitations, including poor mechanical robustness, high sensitivity to humidity, inconsistent surface chemistry, and limited long‐term operational stability [15, 16, 17, 18, 19]. In addition, the relatively low surface charge density and difficulty in achieving controlled micro‐ or nanostructures can restrict their electrical output and scalability for real‐world applications [15]. In contrast, waste thermocol (expanded polystyrene, EPS) represents an abundant and persistent plastic waste stream with intrinsically high tribonegativity and excellent dielectric properties [20].

Waste thermocol, primarily composed of expanded polystyrene (EPS), presents a significant environmental challenge. Widely used in packaging, insulation, and disposable products [21], thermocol is produced in vast quantities but is extremely difficult to degrade [22]. Due to its lightweight and bulky nature, it occupies considerable space in landfills and is prone to scattering, often ending up in waterways and natural ecosystems [23]. Moreover, improper disposal, such as open burning, releases toxic fumes, including styrene vapors and other volatile organic compounds, which are harmful to both human health and the atmosphere [24]. Despite these challenges, thermocol retains significant material value due to its chemical stability and dielectric properties, making it a viable candidate for energy harvesting applications if it can be successfully repurposed or recycled. Polystyrene (thermocol) is known to be highly tribonegative, attributed to its non‐polar chemical structure and the presence of benzene rings, which stabilize surface charges and exhibit high electron affinity [20]. Therefore, transforming this waste stream into a functional nanomaterial for triboelectric energy harvesting presents a promising and sustainable approach to recycling.

The performance of a TENG is not solely determined by the chemical properties of the material; factors such as surface area and dipole alignment also play a critical role due to their direct impact on the surface charge phenomenon [25, 26, 27, 28]. To enhance these properties, researchers have focused on increasing the surface area and optimizing the dipole alignment within the material. One effective approach is electrospinning, a method that has been widely used to synthesize various polymeric nanofibers [25]. Electrospinning improves the surface morphology of the material by creating nanofibers with a significantly higher surface area, which allows for better charge accumulation and transfer [29, 30, 31]. Additionally, the process induces dipole alignment under a high electric field, further enhancing the material's triboelectric properties [26]. This combination of increased surface area and aligned dipoles may leads to improved efficiency in charge generation, thus enhancing the overall performance of the TENG.

Herein, we present a waste‐to‐intelligent‐system strategy in which discarded thermocol is transformed into electrospun EPS nanofibers and employed as an advanced tribonegative layer for high‐performance triboelectric nanogenerators. Although EPS nanofiber‐based TENGs have been previously reported [32], the present work emphasizes nanoscale electronic‐property engineering, where electrospinning‐induced dipole alignment and enhanced electron affinity are experimentally validated, establishing a clear link between nanofiber structure and triboelectric performance. The EPS nanofibers are systematically investigated as a function of device area and operating frequency and are benchmarked against widely used triboelectric polymers under identical conditions, demonstrating their superior output characteristics. Importantly, the long‐term operational durability and environmental robustness of the device are validated through extended aging, humidity, and temperature stability studies, addressing critical challenges for real‐world deployment. Moving beyond conventional energy‐harvesting demonstrations, the developed TENG is further integrated into a self‐powered digital elevator interface, where machine‐learning‐assisted signal processing enables reliable and noise‐resilient human‐machine interaction. This integrated approach elevates recycled EPS nanofibers from a proof‐of‐concept triboelectric material to a scalable platform for intelligent, sustainable, and self‐powered electronic systems.

## 2 Experimental Details

### 2.1 Synthesis of Nanofibers

**FIGURE 1:**
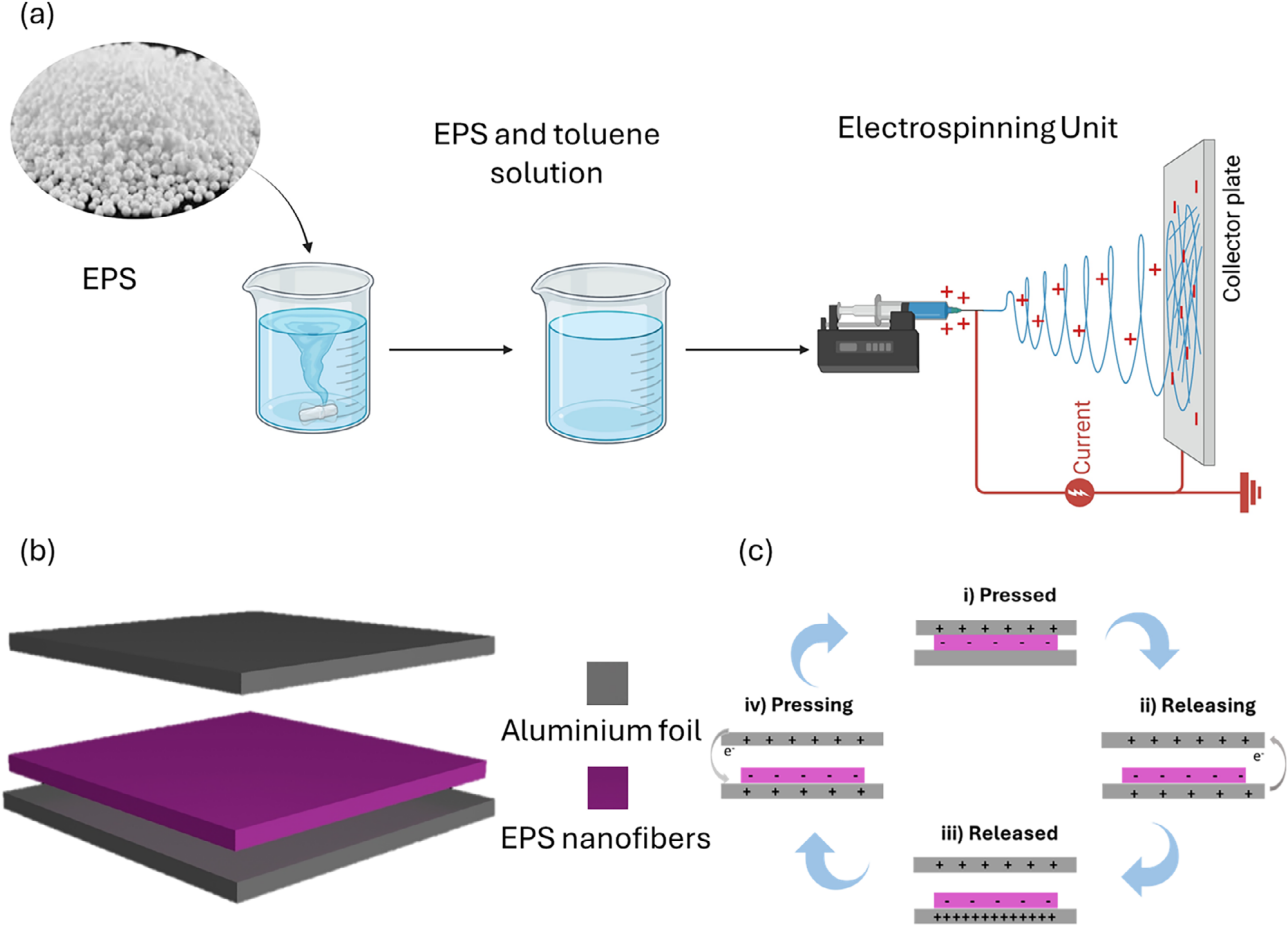
(a) Synthesis of EPS nanofibers. (b) Device structure and (c) mechanism of the EPS nanofibers‐based TENG.

To synthesize the EPS nanofibers, the electrospinning technique was employed, as shown in Figure 1a. First, 0.1 grams of EPS were dissolved in 5 mL of toluene by stirring the mixture at room temperature for 30 min to ensure complete dissolution. Once the solution became transparent, it was transferred into a 1 mL syringe and securely mounted on a syringe pump. The electrospinning setup was configured with a 15 cm distance between the needle and the collection surface. A 20 kV voltage was applied across the system, and the polymer solution was dispensed at a flow rate of 0.2 mL/h. The resulting nanofibers were collected on aluminium foil, which was wrapped around a rectangular collector plate to accumulate the fibers. The concentration variation of the EPS solution has been studied and mentioned in Figure S1.

### 2.2 Device Fabrication

#### 2.2.1 TENG Energy Harvester

The fabricated device operates in a vertical contact‐separation mode. The tribonegative layer consists of electrospun EPS nanofibers deposited onto aluminum foil, which also serves as the bottom electrode. Aluminum foil was used as the tribopositive layer and top electrode due to its high electropositivity and good electrical conductivity. Both triboelectric layers were supported on flexible polyethylene terephthalate (PET) substrates to provide mechanical stability and flexibility. The effective contact area of the device was varied from 4 cm^2^ to 12 cm^2^, with the optimized device having an active area of 12 cm^2^. A small air gap between the two triboelectric layers was maintained to enable repeated contact and separation during mechanical excitation. Figure 1b shows the schematic of the proposed device.

#### 2.2.2 Smart Sensor

For the smart sensor, a strip of aluminium was employed as the bottom triboelectric layer. The EPS nanofiber with aluminium electrode was cut into four small sections and attached to the PET substrate with deliberate spacing between each piece to resemble a switch‐like arrangement. A minimal gap was maintained between the two triboelectric layers to facilitate easy activation upon pressing. The design ensured that contact occurred only in the specific area where pressure was applied, enabling precise, location‐specific sensing. Figure 4 h shows the graphical representation of the smart sensor.

### 2.3 Electrical Measurements

The experimental setup for evaluating the electrical performance of the triboelectric nanogenerator (TENG) consisted of three sequential stages: mechanical excitation, electrical signal measurement, and data acquisition. In the first stage, a programmable linear motor (LinMot HF01‐23) was employed to apply periodic contact–separation forces to the TENG in a controlled and repeatable manner, with the operating frequency and displacement adjusted according to the experimental requirements. In the second stage, the generated electrical signals were measured by directly connecting the TENG electrodes to a digital oscilloscope (DSOIX2012A) to record the open‐circuit voltage and short‐circuit current. In the final stage, the acquired electrical signals were monitored, recorded, and analyzed to evaluate the output performance of the TENG under different operating conditions, including variations in frequency, device area, and external load resistance.

### 2.4 Characterization

The Fourier‐transform infrared (FTIR) spectra of the nanofibers were recorded using a BRUKER ALPHA II instrument to examine their molecular vibrations. Raman spectroscopy was then performed using a Renishaw Raman microscope with a 532 nm laser source to analyze the structural characteristics and disorder within the carbon material. The structural properties of the nanofibers were investigated using a TESCAN MIRA 3 Field Emission‐Scanning Electron Microscope (FE‐SEM), providing insights into their morphology and surface structure. Surface topography was characterized using a non‐contact mode of an NX‐10 AFM (Park Systems, Suwon, Republic of Korea) equipped with a conductive Cr/Pt‐coated probe (Multi 75E‐G, Budget Sensors). Surface potential and work function were analyzed using Kelvin probe force microscopy (KPFM) mode. For KPFM measurements, an AC voltage of 2.5 V at 17 kHz was applied to the tip, while the sample was kept at 0.0 V DC bias. The work function of the sample equation Φs = Φt—eVCPD, where Φt is the work function of the tip, and Vcpd is the measured contact potential difference. The tip work function was calibrated using a gold reference sample with a known work function of 5.2 eV.

### 2.5 Signal Processing and Feature Extraction

The electrical signals generated by the EPS nanofiber‐based TENG were sampled at 100 Hz using an Arduino microcontroller. To transform raw voltage spikes into interpretable commands, a sliding window segmentation was applied with a window size of 20 samples corresponding to 200 ms. For each window, five statistical features were extracted to create a unique signal signature for each floor button: Arithmetic Mean, Standard Deviation, Peak Voltage (*V_max_*), Minimum Voltage, and RMS voltage.

### 2.6 Algorithm and Classification

A Random Forest Classifier was used to perform real‐time classification of the extracted features. This supervised learning model was selected for its ability to discern against noise and to handle non‐linear signal patterns such as those produced by a TENG. The model was trained on a labeled dataset of 200 trigger events (50 taps per floor).

## 3 Results and Discussion

The operating mechanism of the EPS nanofiber–based triboelectric nanogenerator relies on the coupling of contact electrification and electrostatic induction, as illustrated in Figure 1c i–iv. In the fully pressed state (Figure 1c i), the aluminum tribopositive layer comes into intimate contact with the EPS nanofiber tribonegative layer, leading to electron transfer from aluminum to EPS due to their difference in triboelectric polarity, thereby generating opposite surface charges on the two materials while no current flows through the external circuit owing to electrostatic equilibrium. Upon releasing the applied force (Figure 1c ii), the two layers begin to separate, disturbing the charge balance and inducing a potential difference between the electrodes, which drives electrons to flow through the external circuit to compensate for the generated electrostatic field. When the device reaches the fully released state (Figure 1c iii), the separation distance and the corresponding potential difference between the electrodes become maximum, and electron flow continues until a new electrostatic equilibrium is achieved. During the subsequent pressing process (Figure 1c iv), the separation distance decreases, reversing the electric field direction and causing electrons to flow back through the external circuit in the opposite direction. Repeated pressing and releasing cycles under mechanical stimulation thus generate an alternating electrical output, which can be effectively utilized for energy harvesting or self‐powered sensing applications.

**FIGURE 2:**
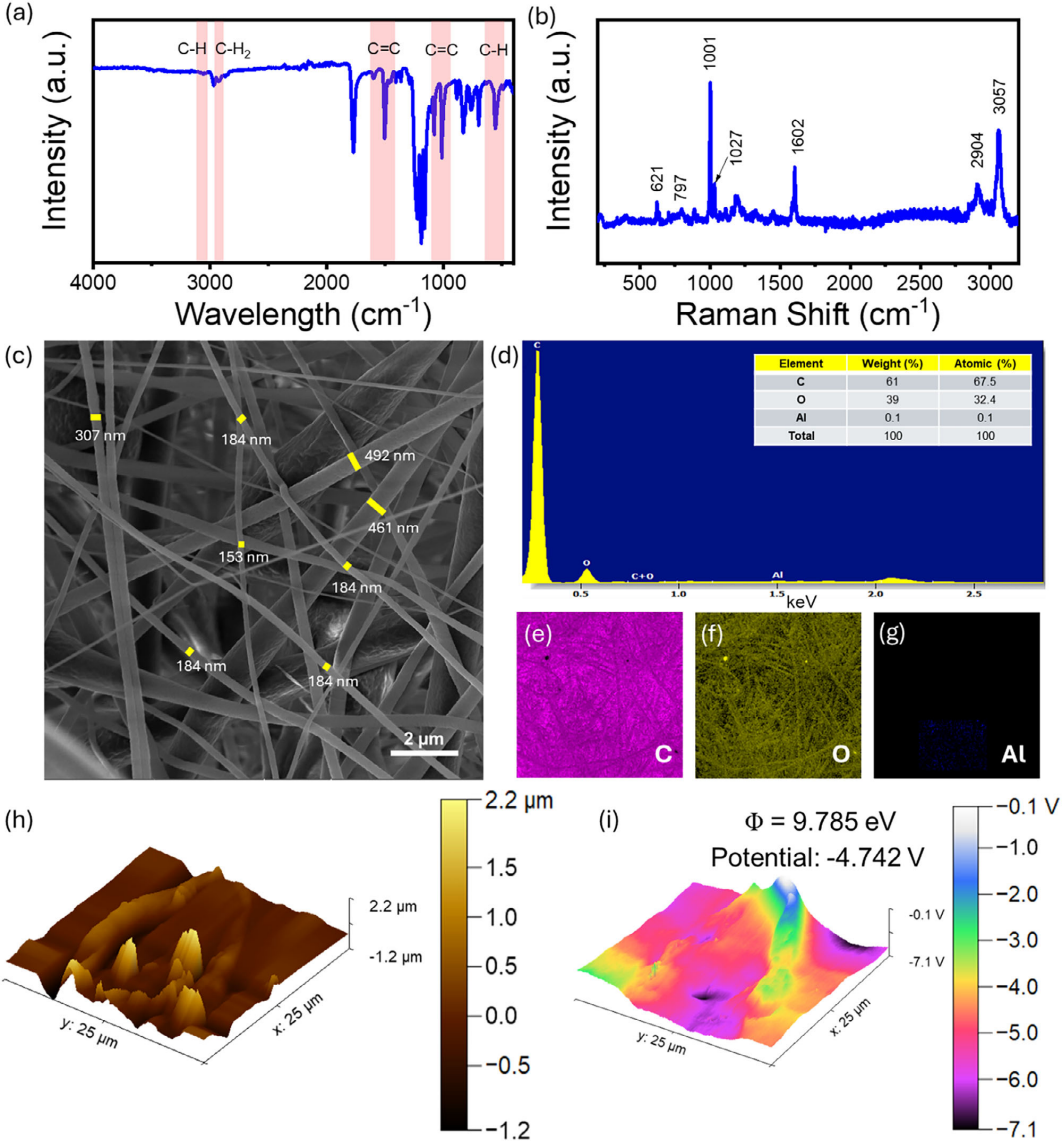
(a) FTIR, (b) Raman, (c) SEM, and (d) elemental analysis of the EPS nanofibers. Elemental mapping image of the (e) Carbon, (f) Oxygen, and (g) Aluminium, (h) AFM 3D surface topography, and (i) KPFM surface potential mapping of the EPS nanofibers.

The EPS nanofibers samples were first analysed by using Fourier‐transform infrared (FTIR) to understand the chemical composition, as shown in Figure 2a. A prominent absorption band at 3055 cm^−^^1^ indicates the presence of aromatic C─H stretching, signifying substituted benzene rings within the polymer matrix. The band at 2925 cm^−^^1^ corresponds to stretching vibrations of methylene groups, pointing to aliphatic chains in the backbone. Further supporting the aromatic composition, bands at 1598 cm^−^^1^, 1504 cm^−^^1^, and 1451 cm^−^^1^ are linked to C═C stretching vibrations typical of benzene ring systems. In the fingerprint region, intense bands at 754 cm^−^^1^ and 706 cm^−^^1^ arise due to out‐of‐plane bending modes of aromatic C─H bonds, a hallmark of polystyrene [20, 33]. These functional groups, especially the electronegative π‐system of the benzene ring and the presence of non‐polar CH and CH_2_ groups, contribute to the tribonegative behavior of EPS [34]. The aromatic rings can stabilize excess electrons through delocalization, making the surface more likely to accept electrons upon contact with other materials. This electron‐accepting tendency under tribological interaction classifies EPS as a tribonegative polymer [35]. The Raman spectrum of EPS exhibits characteristic peaks that confirm its molecular structure and serve as useful markers for material identification, as shown in Figure 2b. In the recorded spectrum, peaks were observed at 621 cm^−^^1^, 797 cm^−^^1^, 1001 cm^−^^1^, 1027 cm^−^^1^, 1062 cm^−^^1^, and 3057 cm^−^^1^. The band at 621 cm^−^^1^ corresponds to out‐of‐plane C–H bending, while the 797 cm^−^^1^ peak arises from in‐plane C–H bending of the aromatic ring. The intense peak at 1001 cm^−^^1^ is attributed to the ring breathing mode of the phenyl group, commonly used as a Raman calibration standard. Peaks at 1027 cm^−1^ and 1062 cm^−^^1^ are associated with aromatic C–C stretching and C–H in‐plane bending vibrations, respectively. The high‐frequency peak at 3057 cm^−^^1^ corresponds to aromatic C–H stretching [36].

The SEM images of EPS nanofibers are shown in Figure 2c. The EPS nanofibers exhibited a well‐interconnected network with an average fiber diameter of approximately 200 nm. Some fibers appeared thicker, likely due to agglomeration during the electrospinning process. Elemental analysis was also performed, revealing a composition primarily of carbon and oxygen, with trace amounts of aluminum, as shown in Figure 2d. The presence of aluminum is presumed to originate from the fiber collection substrate. Figure 2e–g demonstrates the elemental mapping, which confirmed the uniform distribution of carbon and oxygen throughout the nanofiber mat, indicating homogeneity in composition. The surface topography and electronic potential distribution of the synthesized tribonegative material were examined using atomic force microscopy (AFM) and Kelvin probe force microscopy (KPFM). The AFM 3D image (Figure 2h) reveals a rough and heterogeneous surface morphology with nanoscale protrusions and valleys, which are beneficial for triboelectric charge generation as they enhance the effective contact area during frictional interactions. The measured height variations range from approximately –1.2 µm to +2.2 µm, indicating significant surface roughness that can facilitate efficient charge separation and trapping. The corresponding KPFM surface potential mapping (Figure 2i) provides direct insight into the electronic properties of the material. The measured work function is 9.785 eV, and the average surface potential is –4.742 V, which are both considerably higher than those of commonly used tribonegative materials such as polytetrafluoroethylene (PTFE, ∼6.71 eV) [37]. This large work function difference suggests a strong electron affinity of the EPS nanofibers, which can maximize electron transfer when paired with electropositive counterparts in a TENG device. The elevated work function of the EPS nanofibers can be attributed to the aligned dipoles induced during the electrospinning process, enhancing their triboelectric performance. The surface potential distribution spans from –7.1 V to –0.1 V, reflecting spatial heterogeneity that may arise from localized variations in surface states. Such a high work function, combined with nanoscale roughness, underscores the superior suitability of this material as an efficient tribonegative layer for TENG.

**FIGURE 3:**
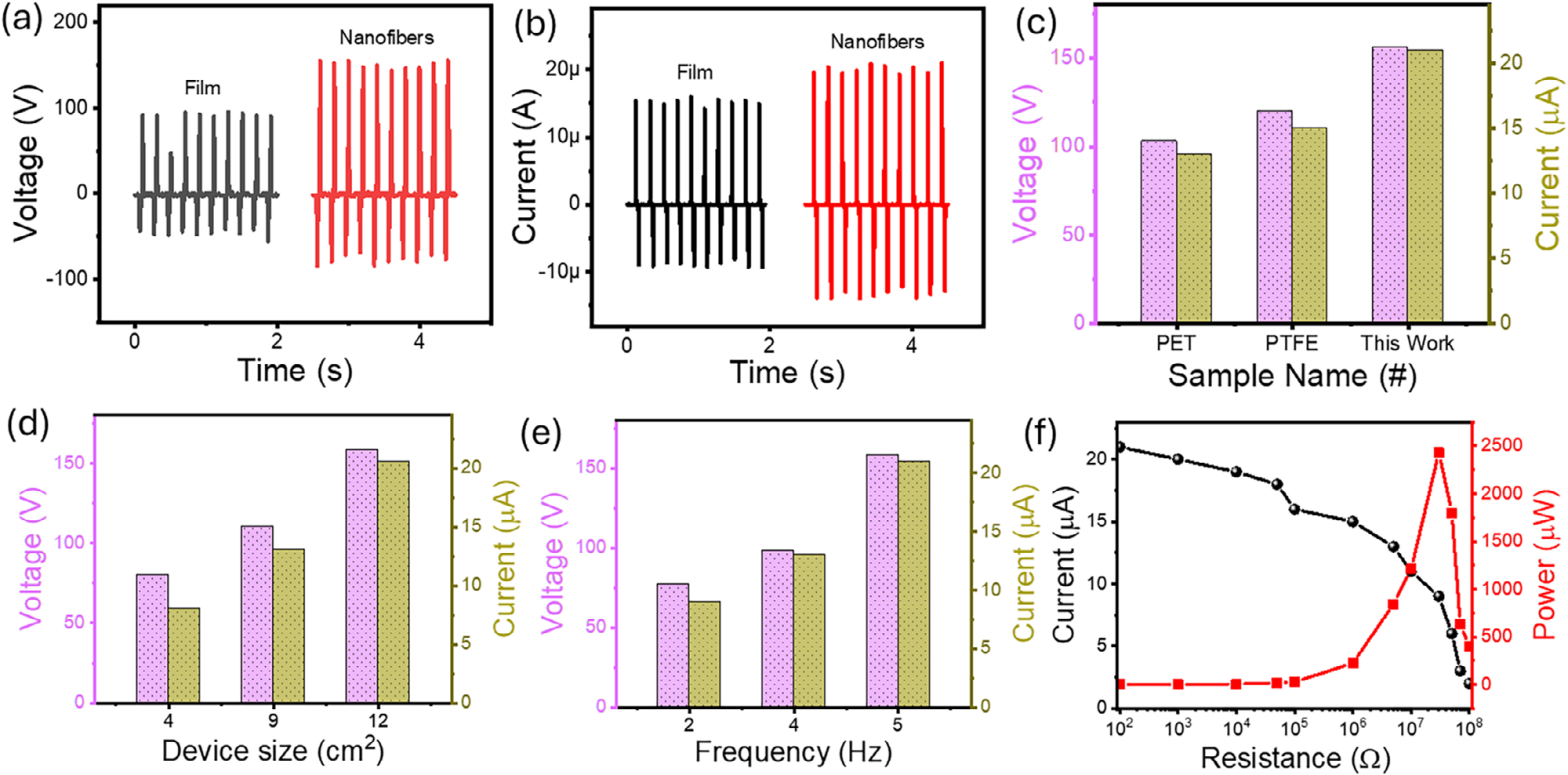
The output (a) voltage and (b) current comparison of the film and fiber‐based EPS TENG. (c) The output comparison of the EPS nanofibers TENG with various commercial polymers. The parameter optimization, such as (d) device size and (e) frequency of the EPS nanofibers based TENG. (f) Instantaneous power of the EPS nanofibers TENG.

The electrical performance of the developed contact‐separation‐based TENG was systematically evaluated by varying the electronegative layers, device dimensions, and applied frequencies, as illustrated in Figure 3. Initially, the open‐circuit voltage and short‐circuit current were measured using aluminium foil as the tribopositive material, paired with either polymer film or nanofibers as the electronegative layers (Figure 3a,b). The film‐based TENG generated a voltage of 100 V and a current of 15 µA. In contrast, the nanofiber‐based TENG exhibited enhanced performance, achieving 160 V and 22 µA. This enhancement is attributed to the larger effective contact area, increased surface roughness, and dipole alignment within the electrospun nanofibers, which collectively improve charge density and energy conversion efficiency [30, 31]. Further investigations involved comparing various electronegative materials, including polyethylene terephthalate (PET), polydimethylsiloxane (PDMS), polytetrafluoroethylene (PTFE), and EPS nanofibers, as shown in Figure 3c. Among the tested combinations, the aluminium‐PET pair generated a voltage output of 103 V and current of 13 µA, followed by aluminium‐PTFE with 120 V and 15 µA. Notably, the aluminium‐EPS nanofiber configuration produced the highest output voltage of 160 V and current of 22 µA, indicating EPS nanofibers exhibit greater tribonegativity compared to the other commercial polymers. This improved performance is attributed to the high surface area and nanoscale roughness of the electrospun fibers, which increase effective contact and enhance triboelectric interactions [38]. In addition, the performance of the TENG was optimized by varying the device area and operating frequency, as presented in Figure 3d,e. An increasing trend in output voltage and current was observed with larger device sizes from 4 cm^2^ to 12 cm^2^, which is attributed to the greater contact area during the triboelectrification process, allowing for more effective charge transfer. Similarly, increasing the frequency from 2 Hz to 5 Hz resulted in improved voltage and current output, due to more frequent contact‐separation cycles that enhance charge accumulation. These results align with the fundamental operating mechanism of TENGs, which generate electricity through the coupling of contact electrification and electrostatic induction driven by periodic mechanical motion. Furthermore, the optimized device configuration comprising an aluminum/EPS nanofiber pair, a 12 cm^2^ active area, and an operating frequency of 5 Hz was employed to investigate the instantaneous output power characteristics of the TENG. As shown in Figure 3f, the output current exhibited a decreasing trend with increasing external load resistance, which is consistent with Ohm's law [39]. In contrast, the output power initially increased with rising resistance, reaching a peak at 30 MΩ, beyond which the power output began to decline. The maximum power output was recorded at 2083 mW/m^2^ for the 30 MΩ load, indicating the optimal matching condition between the internal impedance of the device and the external load. This behavior reflects the typical power transfer characteristics of TENGs, where maximum power is delivered when the load resistance is approximately equal to the device's internal resistance.

**FIGURE 4:**
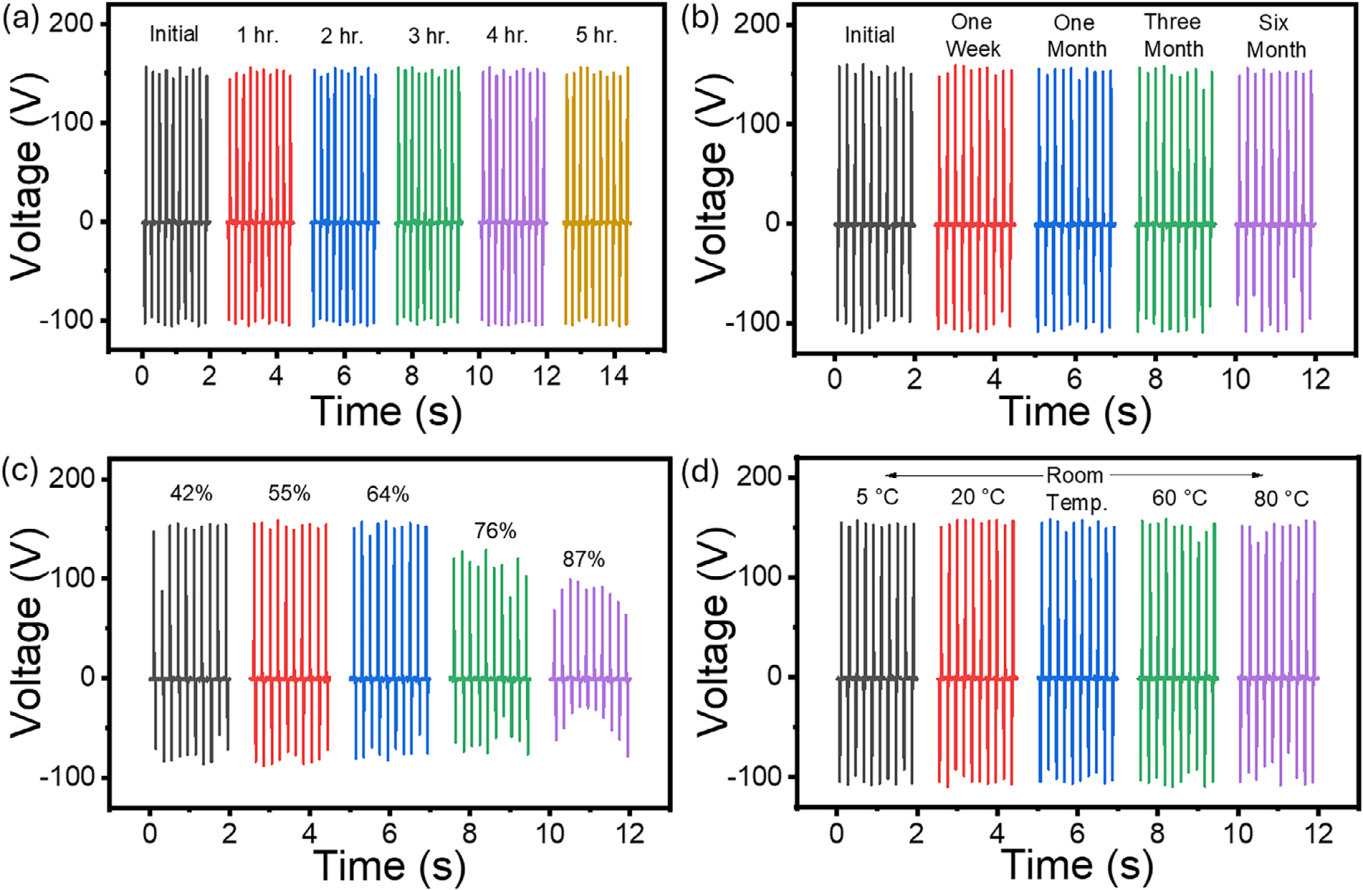
long‐term stability and environmental robustness of EPS nanofibers based on TENG. (a) Long‐term continuous stability, (b) long‐term aging stability, and the effect of (c) humidity, and (d) temperature on the device.

Moreover, the long‐term stability and environmental robustness of the TENG were systematically evaluated to assess its viability for practical, real‐world applications. These tests are crucial because TENGs, when used as energy harvesters or sensors, are often exposed to varying environmental conditions over extended periods. The stability of the optimized device was first assessed under continuous operation for 5 h, with output data collected at one‐hour intervals. As shown in Figure 4a, the device maintained stable electrical performance throughout the test, enduring up to 90,000 continuous contact‐separation cycles without significant degradation, indicating excellent mechanical and electrical durability. In addition to short‐term testing, long‐term aging studies were conducted by operating the device periodically over six months, as depicted in Figure 4b. Performance measurements were taken on a weekly, monthly, quarterly, and semesterly basis. The device consistently retained its output performance, demonstrating high durability and negligible degradation even after prolonged exposure to ambient conditions. Environmental effects such as humidity and temperature were also investigated, as these factors can significantly influence triboelectric performance. Humidity tests were carried out by exposing the device to varying relative humidity (RH) levels, from ambient (∼40% RH) to high humidity conditions (up to 90% RH). The device remained stable up to 70% RH, beyond which a gradual decline in output was observed (Figure 4c). This decrease may be attributed to the absorption of moisture, leading to charge leakage or the formation of a conductive water film between the triboelectric layers. Additionally, aluminum, being a reactive metal, may partially oxidize or react with water at high humidity levels, further affecting performance [40, 41]. Temperature tolerance was evaluated by subjecting the device to both low and high temperatures. For low‐temperature testing, the device was cooled in a refrigerator before being operated using a linear motor. For high‐temperature testing, the device was placed in a vacuum oven for one hour and tested again after reaching thermal equilibrium. As shown in Figure 4d, the TENG exhibited robust and stable output under all temperature conditions from 5°C to 80°C. Interestingly, improved performance was observed at low temperatures, which may be due to enhanced charge retention and more effective triboelectrification at reduced thermal agitation. At high temperatures, the stable performance may result from the evaporation of any residual moisture, improving surface charge stability. These findings confirm the excellent operational stability, environmental tolerance, and long‐term durability of the proposed TENG, highlighting its strong potential for deployment in real‐time and outdoor energy harvesting or sensing applications, even under fluctuating environmental conditions. A quantitative comparison with representative recent EPS and waste‐based TENGs is summarized in Table S1, further highlighting the enhanced output, durability, and functional integration achieved in this work.

For practical deployment in real‐world applications, the proposed TENG can be integrated into a compact and robust packaging structure. A flexible encapsulation strategy using polymeric materials such as PET, polyurethane, or thin PDMS layers can be employed to protect the device from environmental factors, including dust, moisture, and mechanical damage, while preserving device flexibility. The triboelectric contact region can be selectively exposed or covered with a breathable protective layer to ensure effective contact‐separation without significantly compromising electrical output. For applications such as smart switches or human‐machine interfaces, the TENG can be assembled in a layered or modular package, where the active triboelectric layers are enclosed within a protective housing and interfaced with external electronics through sealed electrical contacts. Such a packaging approach would enable scalable fabrication, long‐term reliability, and seamless integration of the TENG into existing infrastructure, thereby facilitating its translation from laboratory demonstrations to practical, self‐powered systems.

**FIGURE 5:**
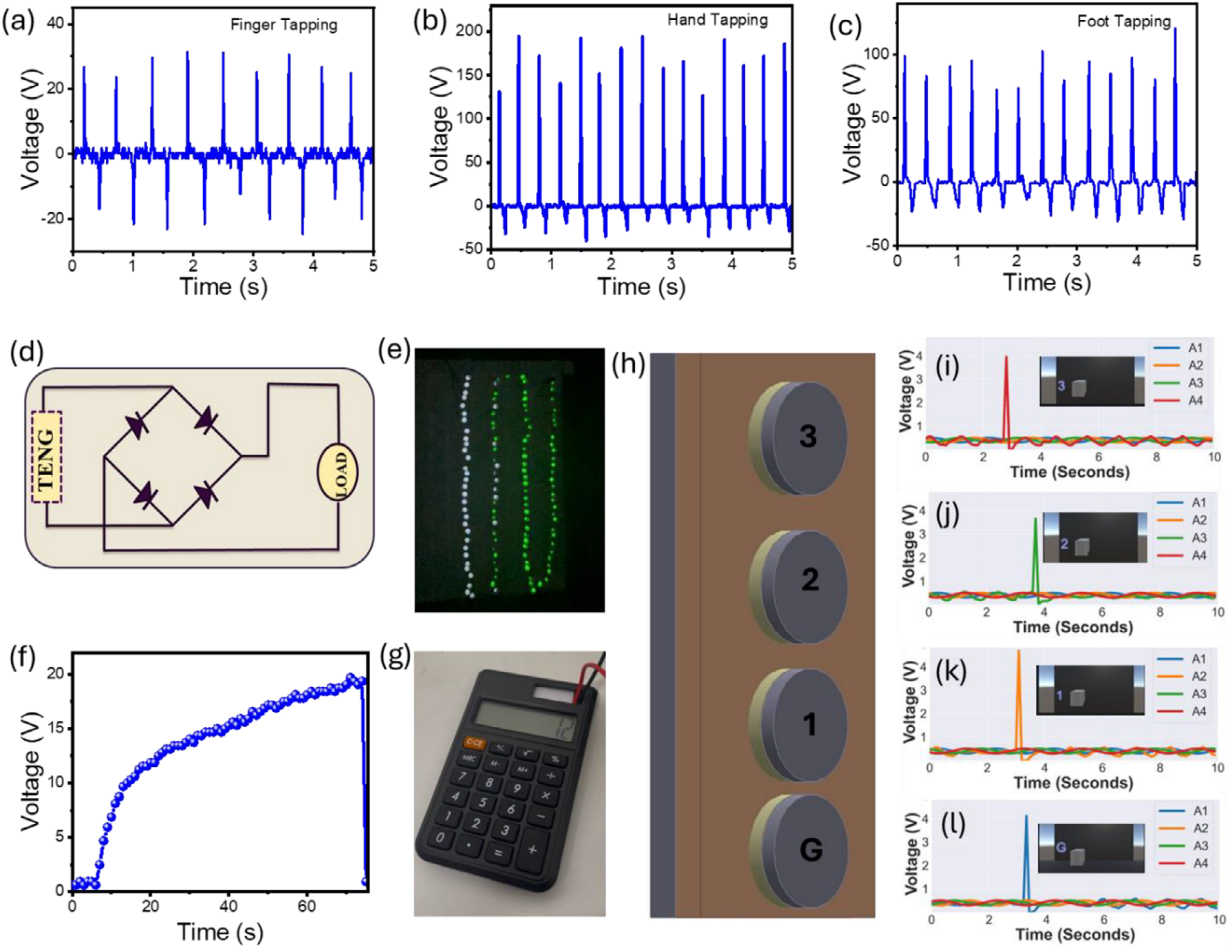
Real‐time applicability of the EPS nanofibers‐based TENG. The voltage generation ability of the TENG is shown by applying the (a) finger tapping, (b) hand tapping, and (c) foot tapping. (d) Circuit diagram for operating LEDs and (e) pictorial image of the LED lit up. (f) Capacitor charging and (g) calculator operation image. (h) The device structure of the smart sensor. The sensor detection of (i) the third floor, (j) the second floor, (k) the first floor, and (l) the ground floor of the digital elevator.

The real‐time applicability of the TENG is demonstrated in Figure 5. Initially, the device was tested under various mechanical stimulations, including finger tapping, hand tapping, and foot tapping, which generated output voltages of approximately 50 V, 180 V, and 130 V, respectively (Figure 5a–c). The observed differences in voltage output can be attributed to variations in contact area, applied force, and pressure distribution during each stimulation. Finger tapping involves a small contact area and lower applied force, resulting in the lowest voltage, while hand tapping covers a larger area with higher force, producing the highest voltage. Foot tapping, despite involving greater overall force, has a more distributed pressure and less effective contact geometry, leading to slightly lower voltage than hand tapping. The device used for these tests had an active area of 12 cm^2^, highlighting that both contact mechanics and applied pressure play a significant role in the TENG's performance. Encouraged by these promising results, the device was further employed to power low‐power electronic devices such as LEDs and a calculator. As most electronic devices require a DC power supply while TENGs inherently generate alternating current (AC), a bridge rectifier was used to convert the AC output into DC [12] as shown in Figure 5d. Using this rectified output, the device successfully powered 80 LEDs through hand tapping, as shown in Figure 5e. Furthermore, to operate a calculator that requires a stable and continuous power supply, a 0.1 µF capacitor was introduced between the rectifier and the calculator to store and regulate the energy. The device efficiently charged the capacitor up to 20 V (Figure 5f), which was then used to power the calculator, enabling it to perform calculations successfully, as depicted in Figure 5g. Inspired by tactile‐triggered energy output, the device was also explored as a potential smart sensor for interactive systems. One such conceptual application involved using TENGs to operate an elevator system. The idea was to utilize the instantaneous voltage spikes generated by mechanical input (e.g., tapping) as input signals to trigger elevator movement. The digital demonstration of this application is shown in Figure 5h–l. For this, the Aluminium strip is connected to the grounded base and four EPS nanofibers with an aluminium electrode connected through a diode and a 10 kΩ resistor, then interfaced with four separate analog pins of an Arduino microcontroller. A simple elevator model was developed using Unity 3D, and real‐time voltage readings from the Arduino were fed into the model using the Arduino IDE. A logical condition was implemented in Unity: if the voltage from any device was at least twice the previous value, it would register as a valid input and trigger the elevator to move to the corresponding floor. The integration of the Random Forest framework significantly enhances the robustness of the digital switching compared to the initial threshold‐based logic. By analysing the unique statistical signatures of each input rather than just the voltage amplitude. This ML algorithm helped filter out noise and ensured only deliberate taps were interpreted as valid commands. The flowchart of the ML is provided in Figure S2. As illustrated in Figure 5i, when the third‐floor button was tapped, the digital elevator model moved accordingly, and the signal was clearly observed on the interface, with only the third‐floor indicator responding while others remained idle. Similarly, pressing the other devices triggered the elevator to move to the respective floors second floor (Figure 5j), the first floor (Figure 5k), and the basement (Figure 5l). This interactive digital demonstration highlights the potential of TENGs to serve as energy‐autonomous, touch‐sensitive digital switches for smart infrastructure. The elevator application showcases how TENGs can provide not only power but also signal input, merging energy harvesting with sensing. Beyond the demonstrated energy harvesting and smart switching functions, the proposed EPS nanofiber‐based triboelectric nanogenerator shows strong potential for a wide range of self‐powered applications. Owing to its lightweight structure, flexibility, and use of recycled polymer materials, the TENG is well‐suited for integration into human‐machine interfaces, touch‐based control panels, and interactive public infrastructure. The device can also function as a self‐powered mechanical or tactile sensor for monitoring human motion, vibrations, or pressure variations in wearable electronics and smart surfaces. Furthermore, its ability to generate stable electrical signals under low‐frequency mechanical excitation makes it a promising candidate for distributed sensing nodes in Internet of Things (IoT) systems, structural health monitoring, and environmental sensing applications. These potential applications highlight the versatility and scalability of the proposed TENG and provide a foundation for future system‐level integration and optimization.

The wear behavior of the triboelectric layer is an important consideration for long‐term and repetitive operation of TENG devices. In the present design, the contact‐separation interaction occurs between a soft EPS nanofiber mat and an aluminum electrode, which significantly reduces abrasive wear compared to rigid‐rigid material interfaces. The electrospun nanofibrous structure can elastically deform under repeated mechanical loading, effectively distributing localized stress and minimizing permanent surface damage. The excellent electrical stability observed over five hours of continuous contact‐separation cycles and over six months of operation indicates that the EPS nanofiber triboelectric layer maintains its structural integrity and charge‐generation capability. Any minor wear that may occur is expected to primarily involve surface smoothing rather than material removal, which does not significantly affect the triboelectric performance. These results suggest that recycled EPS nanofibers exhibit sufficient wear resistance for practical applications involving frequent mechanical contact, such as self‐powered switches and human‐machine interfaces. Despite the promising performance and sustainability potential of the proposed EPS nanofiber‐based triboelectric nanogenerator, several challenges remain to be addressed for practical implementation. Long‐term mechanical durability of the triboelectric layers under repeated contact‐separation cycles may lead to gradual surface wear and output degradation, while environmental factors such as humidity and temperature can affect charge retention and device stability. In addition, the inherently intermittent and low‐frequency electrical output of the TENG necessitates efficient power management and energy storage strategies for continuous operation in real‐world systems. Future research will therefore focus on improving mechanical robustness through surface engineering and protective encapsulation, enhancing environmental stability, and integrating optimized power management circuits. Moreover, the development of greener or solvent‐free EPS processing routes and the exploration of advanced signal processing or machine‐learning‐assisted sensing strategies could further enhance the sustainability, reliability, and application scope of the proposed TENG.

## 4 Conclusion

In conclusion, this study presents a sustainable approach for converting plastic waste into high‐performance nanofibers for triboelectric nanogenerators. Structural and chemical analyses confirmed the high purity of the recycled EPS nanofibers, while electrospinning enhanced surface morphology and dipole alignment, leading to significantly improved triboelectric performance compared to conventional polymers. The resulting TENG demonstrated high electrical output (159 V), excellent mechanical stability (over 90 000 cycles), and strong resilience under diverse environmental conditions. Beyond energy harvesting, the device successfully powered small electronics and enabled a self‐powered, AI‐assisted digital elevator interface, highlighting its practical applicability. By combining recycled materials, nanotechnology, and intelligent systems, this work establishes a scalable route toward eco‐friendly, self‐sustaining electronic devices and paves the way for integrating waste‐derived nanogenerators into smart infrastructure, IoT, and sustainable energy technologies.

## Author Contributions

**Shubham V. Patil**: Conceptualisation, Methodology, Data curation, Formal analysis, Investigation, Writing – original draft, Writing – review & editing. **Mahesh Y. Chougale**: Conceptualisation, Methodology, Data curation, Formal analysis, Investigation, Writing – original draft, Writing – review & editing. **Thomas Cox**: Data curation, Formal analysis, software, Writing – original draft, Writing – review & editing. **Sourabh Ghode**: Methodology, Formal analysis, Investigation. **Abhishek Kulkarni**: Methodology, Data curation, Formal analysis, Characterizations. **Parth Pandit**: Methodology, Data curation, Formal analysis, Characterizations. **Niroshan Manoharan**: Data curation, Formal analysis, Writing – original draft. **Ajay Pandey**: Supervision, Formal analysis, Writing – review & editing. **Jinho Bae**: Supervision, Formal analysis, Writing – review & editing. **Deepak Dubal**: Conceptualisation, Supervision, Formal analysis, Methodology, Investigation, Writing – original draft, Writing – review & editing.

## Conflicts of Interest

The authors declare no conflict of interest.

## Supporting information




**Supporting File**: smll72919‐sup‐0001‐SuppMat.docx.

## Data Availability

The data that support the findings of this study are available from the corresponding author upon reasonable request.
