# Supplementary material for: Recycled Thermocol Nanofibers Based Smart Triboelectric Nanogenerators for AI‐Assisted Switching
Source: Small. 2026 Feb 18;22(23):e12532. doi: 10.1002/smll.202512532 (PMC13100555; doi:10.1002/smll.202512532)
Supplement: Supplementary file 1 — Supporting File: smll72919‐sup‐0001‐SuppMat.docx. [file SMLL-22-e12532-s001.docx]

**Supporting Information**

**Recycled Thermocol Nanofibers based Smart Triboelectric Nanogenerators for AI-Assisted Switching**

*Shubham V. Patil^1^, Mahesh Y. Chougale^1^*, Thomas Cox^1^, Sourabh Ghode^2^, Abhishek Kulkarni^1^, Parth Pandit^3^, Niroshan Manoharan^1^, Ajay Pandey^3^, Jinho Bae^2^, Deepak Dubal^1^*.*

*^1^ Centre for Materials Science, School of Chemistry and Physics, Queensland University of Technology, Brisbane, Australia.*

*^2^Department of Ocean System Engineering, Jeju National University, 102 Jejudaehakro, Jeju 63243, Korea.*

*^3^ School of Electrical Engineering and Robotics, Queensland University of Technology, Brisbane, Australia.*


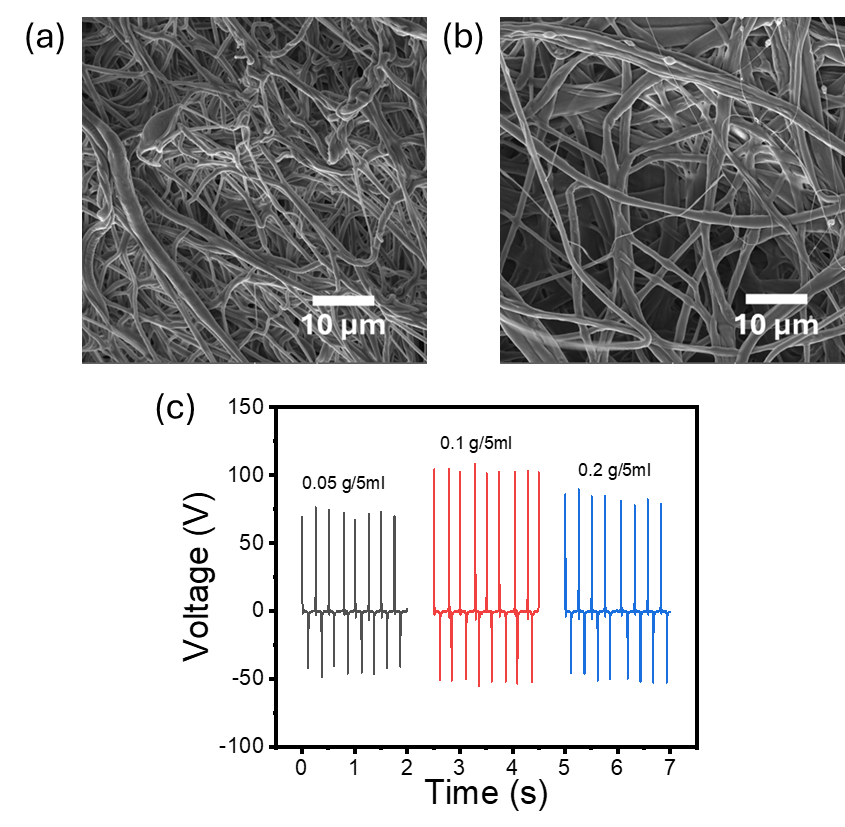


Fig. S1. SEM of the (a) low concentrated and (b) high concentrated EPS nanofibers. (c) comparison results of various concentration of the EPS nanofibers.

The results suggest that the 0.05 g/5 mL EPS solution produces weak fibers with inconsistent thickness as depicted in Fig. S1a, while the highly concentrated 0.2 g/5 mL (Fig. S1b) solution results in thicker fibers. This is reflected in the electrical performance, where the 0.05g/5ml and 0.2 g/5 mL sample shows poorer performance than the 0.1 g/5 mL sample as shown in Fig. S1c.


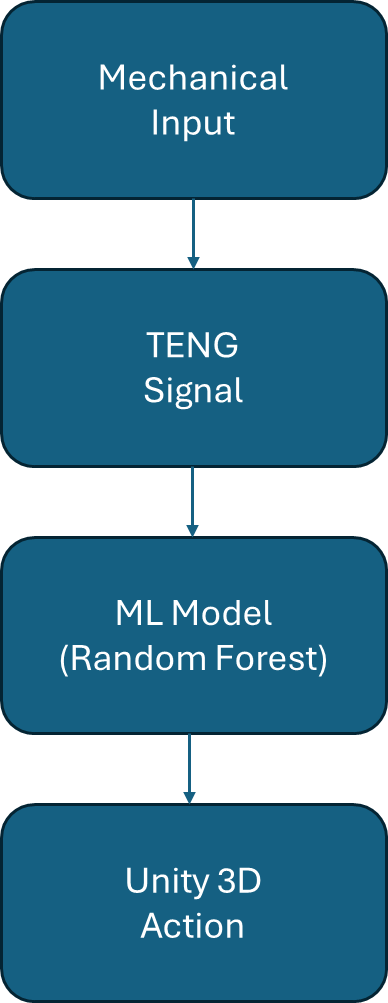


Fig. S2: The 4-step ML process: Mechanical input -> TENG signal -> ML Model (Random Forest) -> Unity 3D action.

**Supporting Table 1:** Comparison of the EPS TENG with previously reported work.

| Materials | Paired material | Frequency/device area | Voltage (V_oc_) | Power density | Ref. |
| --- | --- | --- | --- | --- | --- |
| Petal rose | Poly (methyl methacrylate) (PMMA) | 2 Hz/3 × 3 cm^2^ | 30.06 V | 2.72  µW/cm^2^ | 1 |
| Diatom frustule-chitosan | Fluorinated ethene propene (FEP) | 5 Hz/3 × 4 cm^2^ | 150 V | 1.57  µW/cm^2^ | 2 |
| Egg white (EW) | – | ----/1 × 2 cm^2^ | 55 V | 16.5  µW/cm^2^ | 3 |
| Alginate | Aluminium | 1–4 Hz/5 × 5 cm^2^ | 33 V | 0.38  µW/cm^2^ | 4 |
| Rhododendron leaves | – | 10 Hz/4.5 × 4.5 cm^2^ | 140 V | 15  µW/cm^2^ | 5 |
| EPS nanofibers | **Aluminium** | **5 Hz/4 x 3 cm^2^** | **157 V** | **208 µW/cm^2^** | **This work** |

Based on the comparative data summarized in Table 1, the EPS nanofiber-based TENG developed in this work demonstrates a clear improvement over many recently reported triboelectric nanogenerators employing natural or bio-derived materials. For instance, petal rose-based and alginate-based TENGs exhibit relatively low output voltages of 30.06 V and 33 V, respectively, along with power densities below 3 µW/cm², despite comparable operating frequencies and device sizes. Similarly, egg white-based TENGs show moderate voltage output (55 V) but remain limited in power density. Although diatom frustule-chitosan and rhododendron leaf-based TENGs achieve higher voltages of 150 V and 140 V, respectively, their corresponding power densities remain significantly lower than that of the present EPS nanofiber TENG. In contrast, the EPS nanofiber-aluminum device reported here delivers a high open-circuit voltage of 157 V and a substantially enhanced power density of 208 µW/cm² at a moderate operating frequency of 5 Hz and a compact device area. This superior performance can be attributed to the high surface roughness, enhanced effective contact area, and strong electron affinity of the electrospun EPS nanofibers, highlighting their advantage over conventional bio-derived triboelectric materials. The comparison underscores that recycled EPS nanofibers not only match or exceed the voltage output of similar systems but also offer markedly higher power density, reinforcing their potential for efficient and practical self-powered applications.

References:

1. Y. Chen, Y. Jie, J. Wang, J. Ma, X. Jia, W. Dou, X. Cao, Triboelectrification on natural rose petal for harvesting environmental mechanical energy, Nano Energy 50 (2018) 441–447. <https://doi.org/10.1016/j.nanoen.2018.05.021>.
2. J.N. Kim, J. Lee, T.W. Go, A. Rajabi-Abhari, M. Mahato, J.Y. Park, H. Lee, I.K. Oh, Skin-attachable and biofriendly chitosan-diatom triboelectric nanogenerator, Nano Energy 75 (2020). <https://doi.org/10.1016/j.nanoen.2020.104904>.
3. Slabov, V., Kopyl, S., Soares dos Santos, M. P., & Kholkin, A. L. (2020). Natural and eco-friendly materials for triboelectric energy harvesting. Nano-Micro Letters, 12(1), 42. 10.1007/s40820-020-0373-y.
4. Y. Pang, F. Xi, J. Luo, G. Liu, T. Guo, C. Zhang, An alginate film-based degradable triboelectric nanogenerator, RSC Adv 8 (2018) 6719–6726. <https://doi.org/10.1039/c7ra13294h>.
5. F. Meder, I. Must, A. Sadeghi, A. Mondini, C. Filippeschi, L. Beccai, V. Mattoli, P. Pingue, B. Mazzolai, Energy Conversion at the Cuticle of Living Plants, Adv Funct Mater 28 (2018). <https://doi.org/10.1002/adfm.201806689>.
